# Book Notices

**Published:** 1883-05-20

**Authors:** 


### *BOOK NOTICES.*

A DICTIONARY OF MEDICINE: Including General Pathology, General Therapeutics, Hygiene, and the Diseases Peculiar to Women and Children. By Various Writers. Edited by Richard Quain, M. D., F. R. S:, Fellow and Late Senior Censor of the Royal College of Physicians; Member of the Senate of the University of London: Member of the General Council of Medical Education and Registration; Consulting Physician to the Hospital for Consumption and Diseases of the Chest at Brompton, etc. New York: D. Appleton & Co., 1, 3, 5 Bond Street. 1883.

The above is a large illustrated Dictionary of Medicine of 1,816 double column pages in small, close type in which the author has sought to present "the vast number of facts and observations by which the recent progress of scientific and practical medicine has been marked as diffusely recorded in the transactions of learned societies, in journals and in systematic treatises." The great value and importance of such a work to the profession is at once apparent. The Dictionary of alphebetical method was adopted as best calculated for easy reference. It is a dictionary not so much for definition as for general and detailed information upon almost every topic of importance. A dictionary of medicine rather than that of surgery or Therapeutics, in which diseases are fully treated in alphabetical order.

As an English work, it details the advances in Europe perhaps more fully than in our own country, which, to those desiring the most extended information, will constitue an advantage rather than an objection.

In the preparation of this great work, the editor secured the co-operation of a large number of able and distinguished medical writers, each selected to write on a subject with which he was specially acquainted. We feel safe in recommending this work to our readers as containing a vast amount of important information, much of which is derived from the latest advances in the profession.

